# Y-Chromosome-Linked Genes Are Associated With Sex-Related Head-Neck Squamous Cell Carcinoma Survival

**DOI:** 10.1002/ohn.421

**Published:** 2023-07-07

**Authors:** Xin Feng, Tan Zhang, Jeff Chou, Hafiz S. Patwa, Christopher A. Sullivan, J. Dale Browne

**Affiliations:** 1 Departments of Otolaryngology, Wake Forest School of Medicine, Winston-Salem, North Carolina, USA; 2 Internal Medicine, Section on Gerontology and Geriatric Medicine, Wake Forest School of Medicine, Winston-Salem, North Carolina, USA; 3 Center for Cancer Genomics and Precision Oncology, Wake Forest School of Medicine, Winston-Salem, North Carolina, USA; 4 Department of Cancer Biology, Wake Forest School of Medicine, Winston-Salem, North Carolina, USA

**Keywords:** gender difference, head and neck squamous cell carcinoma (HNSCC), immune response, minor histocompatibility antigen (MHC), overall survival, Y-linked genes

## Abstract

**Objective.:**

To define novel gene biomarkers for prognosis of head and neck squamous cell carcinoma (HNSCC) patients’ survival.

**Study Design.:**

Retrospective study.

**Setting.:**

The Cancer Genome Atlas (TCGA) HNSCC RNA-Seq dataset.

**Methods.:**

Coexpressed gene clusters were extracted from TCGA RNA-seq data using our previously published method (EPIG). Kaplan-Meier estimator was then used for overall survival-relevant analysis, with patients partitioned into 3 groups based on gene expression levels: female, male_low, and male_high.

**Results.:**

Male had better overall survival than female and male with higher expression level of Y-chromosome-linked (Y-linked) genes had significantly better survival than those with lower expression levels. In addition, male with a higher expression level of Y-linked genes showed even better survival when they have a higher level of coexpressed cluster of genes related to B or T cell immune response. Other clinical conditions related to immune responses also consistently showed favorable effects on the Y-linked genes for survival estimation. Male patients with higher expression level of Y-linked genes also have significantly higher tumor/normal tissue (T/N) ratio of those genes and higher level of several immune responses related clinical measurements (eg, lymphocyte and TCR related). Male patients with lower expression level of Y-linked genes benefited from radiation-only treatment.

**Conclusions.:**

The favorable role of a cluster of coexpressed Y-linked genes in HNSCC patients’ survival is potentially associated with elevated level of immune responses. These Y-linked genes could serve as useful prognostic biomarkers for HNSCC patients’ survival estimation and treatment.

Head and neck squamous cell carcinoma (HNSCC) is the most common cancer developed from the mucosal epithelium of the head and neck area and about 900,000 new diagnoses/450,000 death each year worldwide.^1^ HNSCC has been associated with tobacco and alcohol consumptions and human papillomavirus (HPV) infection is another high-risk factor for increasing incidence, especially in the male.^2‐5^ Although male patients have more than twice the incidence of HNSCC compared to female patients, they have a better overall survival than the females.^6-8^ This gender disparity in overall survival may be dependent on the individual patient's age, race, tumor location, stage, HPV status, and treatment.^6,8‐12^ By analyzing TCGA dataset, it was found that mutations in BRWD3 gene in HPV-negative female patients were associated with poorer overall survival.^13^ It is possible that other genes could also be involved in this gender disparity for HNSCC survival and discovery of these genes may further illuminate the underlying mechanisms regulating these gender disparities in HNSCC incidence and prognosis.

Previous study using TCGA data has been devoted to identify network-based gene signature for developing predictors of progression status in HNSCC.^14^ We have recently identified several clusters of coexpressed genes that are associated with HNSCC overall survival by analyzing TCGA dataset.^15^ Briefly, 4 clusters of coexpressed genes were identified, with 2 of them favorable and 2 of them unfavorable to HNSCC overall survival. In addition, their association with survival is independent of other clinical condition, for instance, HPV status or tumor sites. Notably, one of the favorable cluster of genes are Y-linked genes that are located on the nonrecombining region of the human Y-chromosome.^16^ Given that growing evidence are now showing that Y-linked genes are functioning as regulators of gender-specific cancer incidence and prognosis,^17–21^ in this study, we further performed analysis on this cluster of Y-linked genes and examined their role in HNSCC prognosis and gender disparities regarding to overall survival.

The main HNSCC treatments are surgery, radiation, and chemotherapy.^22^ Responses to chemoradiotherapy (CRT) treatment in HNSCC patients could be affected by patients’ biological factors, for example, tumor microenvironment, antitumor immunity, immunosuppression, epigenetic modification, DNA damage, and so on.^23,24^ It is unknown if the Y-chromosome-linked genes could interfere with the response to CRT treatment in HNSCC. The patients from the TCGA database received tumor removal by surgery following with/without CRT. By using those data, we also looked into the treatment response to CRT between the male and female and the male with different Y-linked genes expression levels in HNSCC patients.

## Methods

The data analyzed in this study were from TCGA (http://cancergenome.nih.gov) managed by the National Cancer Institute and National Human Genome Research Institute. Analysis of these public datasets does not require review and exempted by the Institutional Review Board of Wake Forest University Health Sciences. TCGA HNSCC RNA-Seq raw counts were downloaded from https://portal.gdc.cancer.gov/. The normalized expression of each gene of all 499 primary tumor samples was generated using limma-voom.^25^ Limma is an R package for the analysis of gene expression data, which uses linear model to assess differential expression in the context of multifactor-designed experiments. The Y-linked genes favorable to overall survival were extracted by a previously reported method, “extracting gene expression patterns and identifying co-expressed genes” (EPIG).^15,26^ EPIG is an unsupervised cluster extraction method that goes through a pair-wised correlation evaluation among all the genes to extract all the possible clusters with a given correlation threshold. The genes in each of the extracted patterns are highly similar in their expression profiles, which may be caused by coexpression to underlying biological cause. Notably, this extracted cluster is unsupervised, since no predefined profiles or any demographic information, such as gender, race, stages, and so on, were used. We then partitioned tumor samples into female and male with low and high expression groups to determine if this cluster was survival-relevant using Kaplan-Meier estimator. Kaplan-Meier estimator was also used to estimate the survival relevance to the combination of Y-linked cluster with B/T cell immune-related cluster genes and relevance to the treatment response to CRT in the patients with different Y-linked cluster genes expression.

Multivariate cox regression was used to identify the correlation of immune-related factors to the overall survival in HNSCC patients with high and low expression of the Y-linked genes.

A student’s *t* test was used to compare the immune-related factors between male patients with high and low expression of Y-linked genes. The alpha level was set at *p*=0.05.

Pairs of tumor and normal tissue data were obtained from TCGA data and average of all 9 selected genes were used for calculation of tumor to normal (T/N) ratio.

## Results

### Correlation of Y-Linked Cluster of Genes With Overall Survival in HNSCC Patients

**Figure 1.**
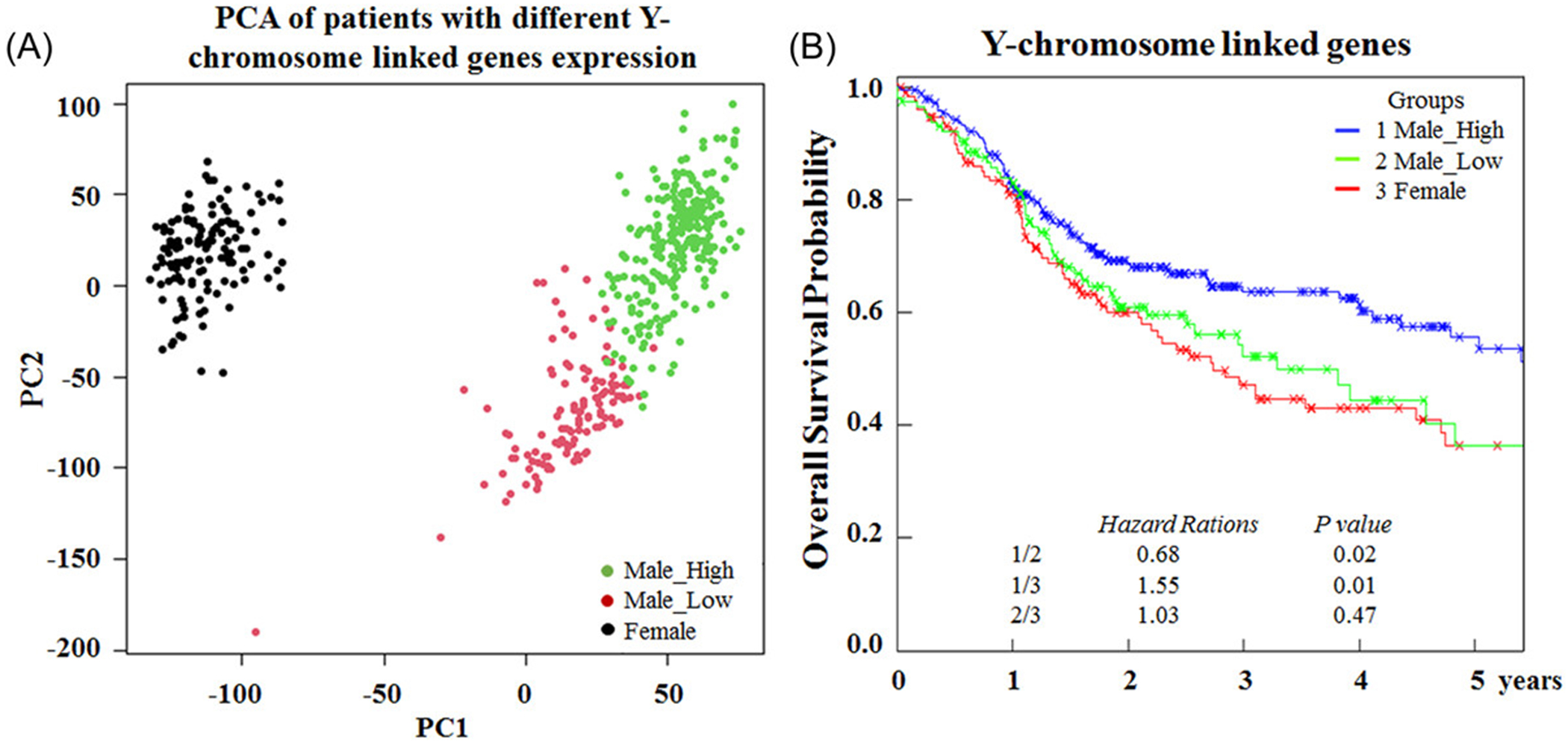
Overall survival comparison between female and male with high and low expression of Y-linked genes in HNSCC patients. (A) PCA plots of Y-linked genes. (B) Comparison between male and female. HNSCC, head and neck squamous cell carcinoma.

Our previous study has reported that Y-linked gene cluster (cluster 1 in our last report^15^) as one of the gene clusters favorably correlated to the overall survival in HNSCC patients. In this study, we set a middle value and separated the male patients into 2 groups (high and low) according to the expression level of Y-linked cluster of genes. The gene expression distribution of these 3 groups of patients (male_high, male_low, and female) is shown in Figure 1A. We found the male patients with high expression of Y-linked cluster of genes had the best overall survival compared to male with low expression and female patients, who do not have any expression of those genes (*p* < 0.05). No significant difference was found between the male with low genes expression and the female patients (Figure 1B).

### Correlation Between Immune-Related Factors and Overall Survival in the Male Patients With Different Expression Levels of Y-Linked Genes

With multivariate cox regression analysis, we identified some immune-related characters which are differently involved in survival depending on the expression levels of Y-linked genes (Table 1). In patients with high expression level of Y-linked genes, overall survival was positively correlated to resting mast cells, lymphocyte infiltration, T follicular helper cells, regulatory T cells, and T cell receptor richness and negatively correlated to activated mast cells and naïve CD4 T cells (*p* < 0.05). In patients with low Y-linked genes expression levels, overall survival was positively correlated to the naïve B cells (*p* < 0.05).

### Effects of Combined Gene Clusters of Y-Linked Genes With B or T Cell Immune-Related Genes on the Overall Survival in HNSCC Patients

**Figure 2.**
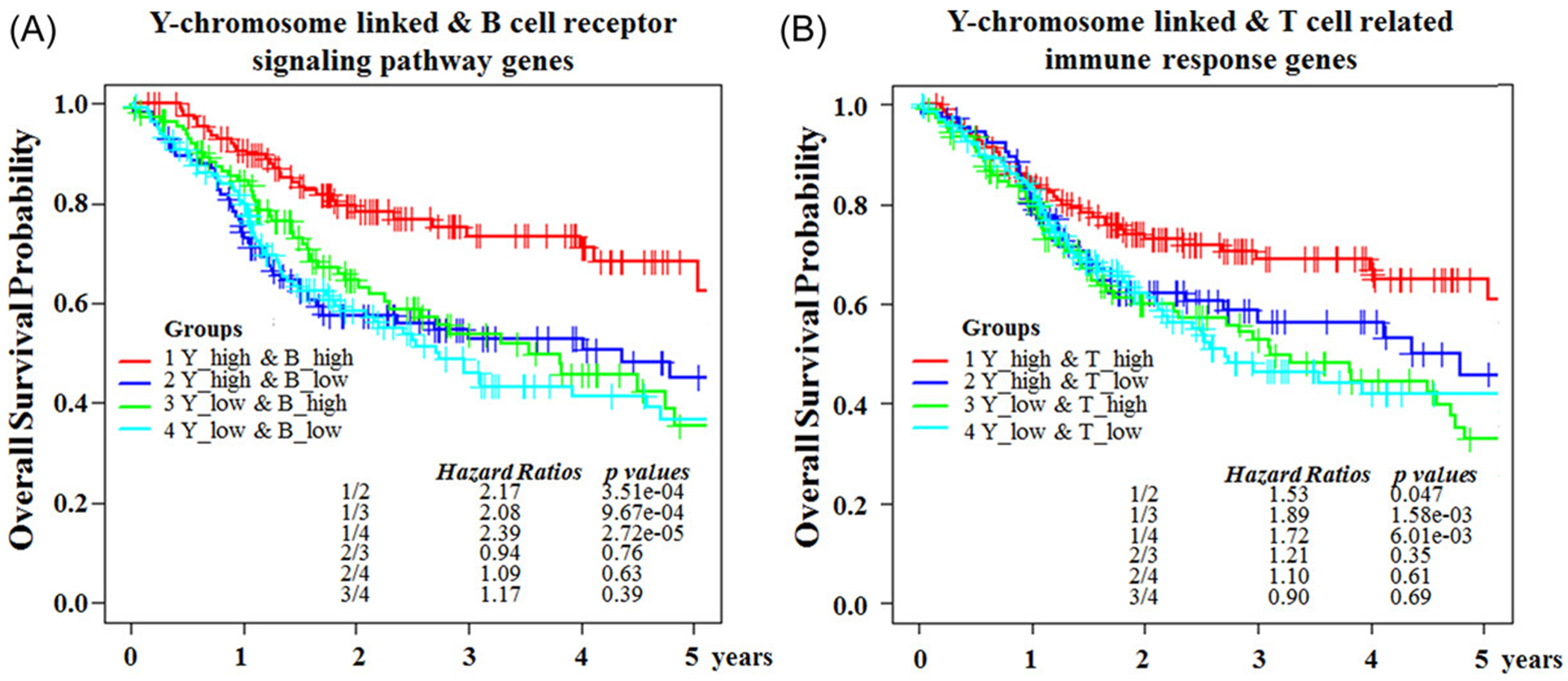
Combination of Y-linked genes with immune-related genes affects overall survival in HNSCC patients. (A) Combine with B immune-related genes. (B) Combine with T immune-related genes. HNSCC, head and neck squamous cell carcinoma.

We got 4 combinations of genes clusters based on the genes expression levels (Y_high + B/T_high, Y_high + B/T_low, Y_low + B/T_high, Y_low + B/T low). The patients with a high Y-linked and high B cell receptor signaling pathway genes cluster (Y_high + B_high, Figure 2A) or T cell-related immune response genes cluster (Y_high + T_high, Figure 2B) had better overall survival compared to all the other 3 combinations.

### Comparison of the T/N ratio Based on the Average Expression of Top 9 Y-Linked Genes

**Figure 3.**
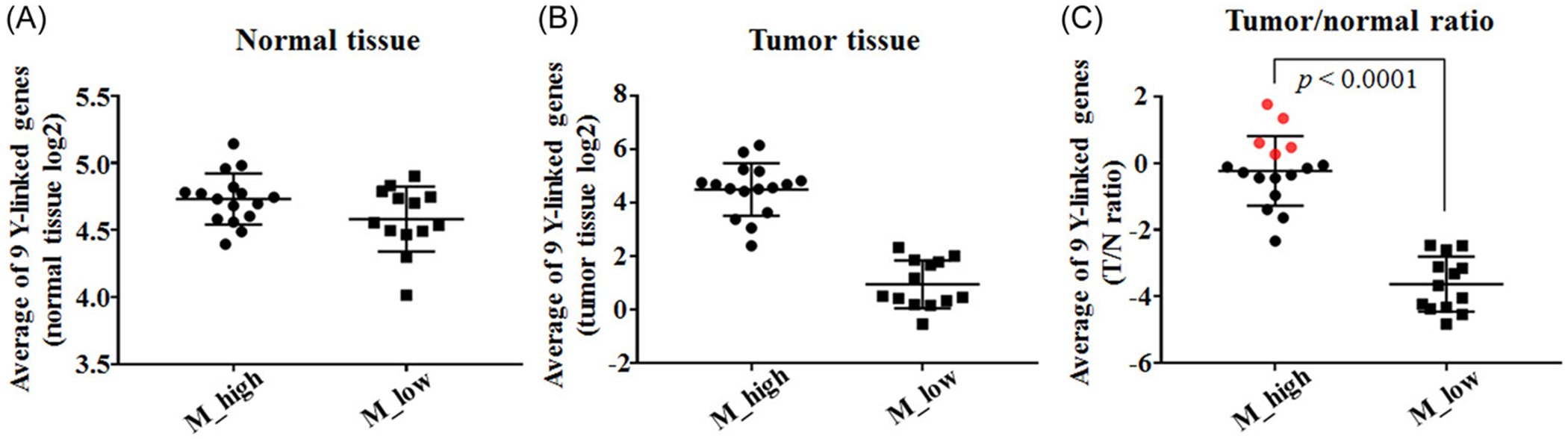
Comparison of expression levels or relative abundance of 9 Y-linked genes between M_high and M_low HNSCC patients. (A) normal tissue, (B) tumor tissue, and (C) T/N ratio. HNSCC, head and neck squamous cell carcinoma; T/N, tumor/normal tissue.

Selected top 9 highly expressed genes among 22 of Y-linked cluster of genes are RPS4Y1, DDX3Y (DBY), KDM5D, USP9Y (DFFRY, SPGFY2), EIF1AY, UTY (KDM6C), TXLNGY, PRKY, and ZFY. Totally 29 male patients were divided into 2 groups with 16 high (M_high) and 13 low (M_low) based on Y-linked gene expression. No significant differences were found within the normal tissues in these 2 groups (Figure 3A) and a lower expression tendency in M_low compared to the M_high was observed (Figure 3B). We found that M_high group showed significantly higher (~10,000 folds, *p* < 0.0001) T/N ratio of 9 gene average expression than the M_low group (Figure 3C).

### Identification of Differences in Some Immune-Related Factors Between HNSCC Patients With High and Low 9-gene T/N Ratios of Y-Linked Genes

**Figure 4.**
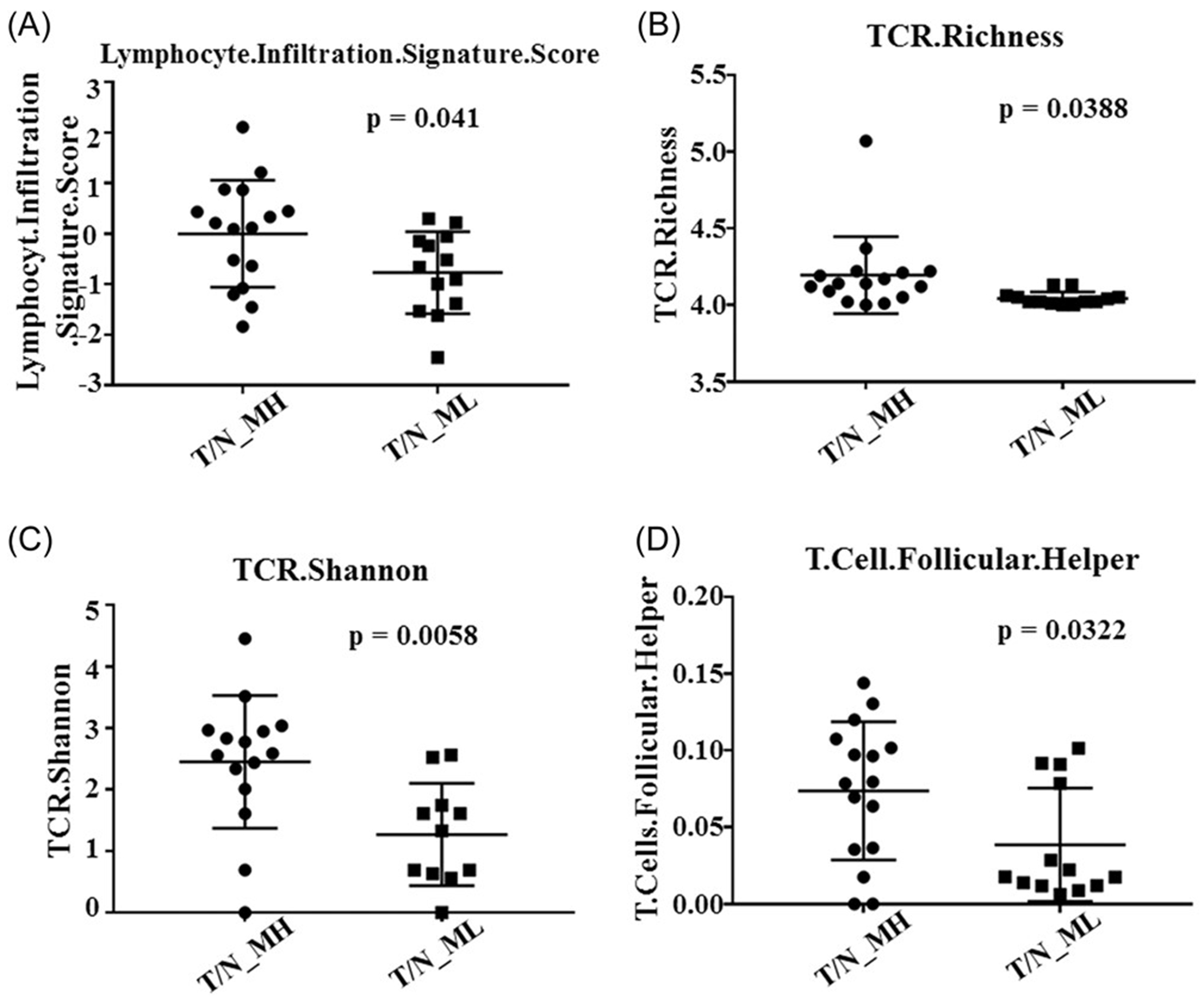
Differences of immune-related biomarkers between 9 Y-linked genes expression T/N ratio high and low groups of male HNSCC patients. (A) Lymphocyte.Infiltraion.Signature.Score. (B) TCR.Richness. (C) TCR.Shannon. (D) Cell.Follicular.Helper. HNSCC, head and neck squamous cell carcinoma; T/N, tumor/normal tissue.

Higher expression of some immune (lymphocyte infiltration, Figure 4A, *p* < 0.05), especially T cell immune-related factors including TCR Richness (Figure 4B, *p* < 0.05), TCR.Shannon (Figure 4C, *p* < 0.01), and T follicular helper cells (Figure 4D, *p* < 0.05) were found in the male patients with high T/N ratio (T/N_MH) than those with low T/N ratio (T/N_ML).

### Survival Relevance to the CRT Treatments in HNSCC Patients With Different Y-Linked Genes Expression

**Figure 5.**
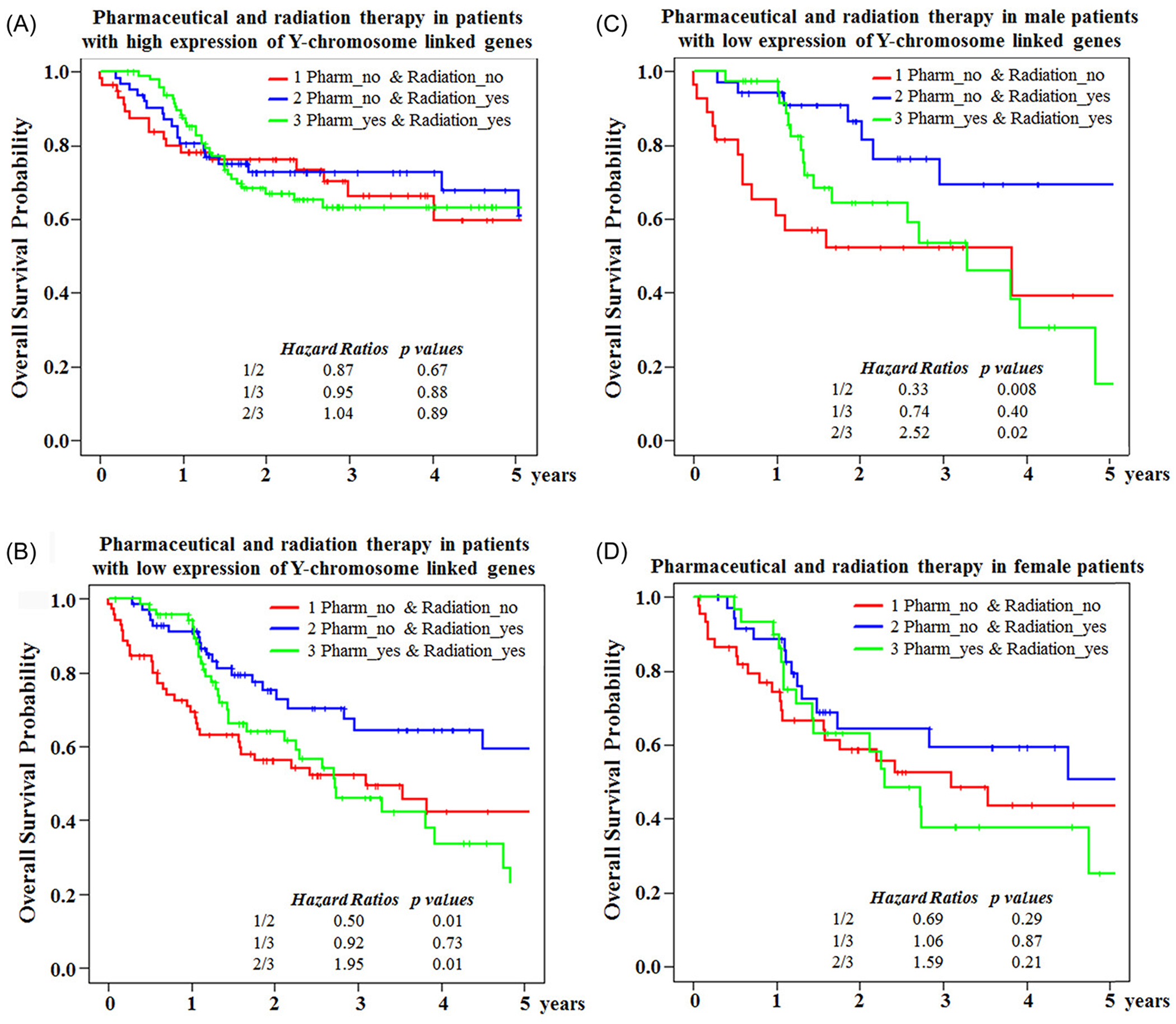
Different response to CRT treatment in patients with different Y-linked genes expression. Patients with high (A) and low (B) Y-linked genes expression. Male patients with low Y-linked genes (C) and female patients (D). CRT, chemoradiotherapy.

The overall survival in patients with the high expression level of Y-linked genes were similar and not affected by treatments (radiation-only, radiation plus pharmaceutical, no pharmaceutical, and no radiation) (Figure 5A). However, in patients with low expression level of Y-chromosome-linked genes, radiation-only treatment had better overall survival than radiation plus pharmaceutical therapy (*p* < 0.05) and no treatments (*p* < 0.01) (Figure 5B). Given that patients with low expression of Y-linked genes in Figure 5B included both male and female, we further separated genders and found that male patients with low expression level of Y-linked genes showed same differences as were found in Figure 5B and C. However, no significance of overall survival to different treatments were found in the female patients (*p* > 0.05, Figure 5D).

## Discussion

Our study revealed that the overall survival of HNSCC was correlated to the Y-linked cluster of genes, which is consistent to the finding on an association of loss of Y-chromosome with poor prognosis in male HNSCC.^21^ Specifically, female and male with lower expression levels of these Y-linked genes have a significantly lower overall survival rate than male with higher expression levels of these genes. In addition, male patients with high Y-linked gene expression and high level of immune response-related factor showed the best overall survival. Our finding strongly suggest a novel role of these Y-linked genes and immune microenvironments in regulating HNSCC progress and prognosis.

The Y-linked genes in this study belong to X-Y homologous genes and the expression of these genes are close to zero in the female. The amount of Y-linked genes was differently expressed in the male patients and patients with high gene expression showed high overall survival in HNSCC. By further examining available TCGA clinical conditions, we found that in the high Y-linked genes expression patients, the overall survival is positively correlated to the antitumor immune response. For instance, lymphocyte infiltration, high resting mast cells, low activated mast cells, and T cell immune response (eg, the regulatory T cells, follicular helper T cells, low naïve T cells, and TCR richness). However, in the Y-linked genes low expression patients, we only found that high naïve B cells were positively correlated to overall survival. Lymphocyte infiltration,^27,28^ mast cells,^29,30^ T regulatory, and T helper cells^31^ are important components of the innate immune system and their functions and modulations have been well studied in tumor development, angiogenesis for tumor progression/metastasis, antitumor immune response, and prognosis in HNSCC.^32^ TCR repertoire is also well known as a novel indicator for immune monitoring and for its correlation with therapeutic response and prognosis prediction in cancers.^28,33^ Notably, the above-mentioned immune response-related factors were correlated to overall survival of HNSCC only in the patients with higher expression levels of the Y-linked cluster of genes. Whether this cluster of genes encoded proteins cooperate with or activate these immune-related factors/responses in the HNSCC needs further studies to determine.

Both B cell and T cell immune responses are known to play a key role in regulating the progress and prognosis of HNSCC.^34-37^ We have also recently reported that B cell immune-related cluster of coexpressed genes are favorably associated with HNSCC overall survival.^15^ It was also reported that CT8^+^ T cell-related 8-genes signature could play an effective role in HNSCC prognosis prediction and immunotherapy response.^38^ We further found that patients with high expression of Y-linked genes had significant better overall survival only when in combination with high B cell receptor signaling pathways related cluster of genes or high T cell immune response-related cluster of genes. This result suggested that Y-linked genes may have an effect on the immune responses during HNSCC tumor prognosis and treatment. Further study to explore the mechanism underlying the modulation between Y-linked and T/B cell immune-related genes and proteins may lead to the development of high accuracy and efficiency biomarkers for HNSCC prognosis and treatment responses.

To better compare the relative abundance of Y-linked gene expression in HNSCC patients, we analyzed male patients with paired normal and tumor tissues individually. We found that patients with high Y-linked gene expression have significantly higher T/N ratio of average 9 top genes expression compared to the low expression group. This finding strongly support a potential use of this T/N ratio in the prediction of HNSCC survival. On the one hand, the T/N ratio measurement will avoid the variance of gene expression from normal tissue and tumor tissue cross individuals. On the other hand, the use of T/N ratio value will also avoid incomparability when estimating gene expression levels using data collected by different techniques/platforms or from different labs. Consistently, among top 9 Y-linked genes, KDM5D and UTY have been reported as tumor suppressors and predictors for better prognosis in head and neck cancers and other tumors.^17,18,39–41^ In addition to their tumor suppression effects, KDM5D, UTY, together with DDX3Y, USP9Y, RPS4Y1, and EIF1AY, are also known as genes that encode male-specific minor histocompatibility antigens (mHAs).^42–45^ It will be interesting to see if there are elevated mHA protein expression in HNSCC tumor and their respective targeting antibodies in the circulation of HNSCC patients, as were found in graft-versus-host diseases.^46,47^

With Kaplan-Meier tool,^14^ we found that the mean expression of the above 9 Y-linked genes are positively associated with patients survival of bladder carcinoma, pancreatic ductal adenocarcinoma, rectum adenocarcinoma, and thymoma similarly to HNSCC, but negatively associated with survival of kidney renal papillary cell carcinoma and stomach adenocarcinoma (Supplementary Table S1, online available). It suggested that these Y-linked genes could also act as predictors for progression and prognosis in these cancers.

Interestingly, we further found that male patients with high Y-linked gene expression has good prognosis independent to either pharmaceutical (ie, chemotherapy) or radiation therapy. However, for those male patients with low Y-linked genes had worse prognosis, the radiation-only treatment can improve survival but no benefits of radiation plus pharmaceutical treatment on survival was found. It was reported that patients’ treatment response to CRT in HNSCC are various according to patients’ biological determinants and tumor pathological characteristics.^22,23^ For example, tumorinfiltrating lymphocyte favor the response to CRT of the head and neck cancer^48^ and tumors with loss of Y-chromosome exhibited overexpression of some genes implicated in resistance to CRT in HNSCC.^21^ HNSCC is considered as an immunosuppressive disease^49^ and immunotherapy has been introduced into HNSCC treatment. For instance, the immune checkpoint inhibitor for recurrent or metastatic HNSCC.^50,51^ However, not all patients are selected to accept immunotherapy as the first-line treatment and one-third or even less of unselected patients showed response to check point inhibitor.^51^ Efficacy and toxicity of immune therapies are still under evaluation.^52^ It is important to understand the modulation of the immune system by itself during disease development and its response to all the treatments including immunotherapy. Our findings support a possible role of Y-linked genes in the modulation of the immune system during various treatments and develop strategies to improve responses to HNSCC treatment. It also suggests that the Y-linked genes might be valuable indicators for future guidance on making personalized treatment strategy, avoiding overtreatment, and reducing medical cost and side effects from CRT in HNSCC patients.

In summary, our study found the Y-linked cluster of genes are favorably associated with the overall survival in HNSCC patients when expressed higher in the tumor. Until now, HPV status with combined positive score (locations, stages, response to the treatments) is the only consistently used predictive biomarker for patients with oropharyngeal cancer.^53^ Lacking of reliable biomarkers for prognosis and treatment response prediction brings difficulties for clinical doctors in making an ideal treatment plan. This study is the first step of our research goal to find novel effective and reliable biomarkers. Importantly, the identified predictive value of Y-linked genes were independent on any predefined clinical profiles or demographic information, such as race, stage, HPV status, tumor sites, and so on. Making these genes an ideal candidate as novel biomarkers that could be easily applied for clinical use. We admit that it is hard to determine the advantage of our identified genetic profile over other markers at this stage. Future cohort study is also needed to further explore the role of these Y-linked genes in regulation of host immune status. Innovative therapy strategy based on these gene clusters could also be developed for precision medicine.

## Conclusion

Our work suggest that a cluster of Y-linked genes are coexpressed in HNSCC tumors and may play a role in survival disparity regulation between male and female HNSCC patients, which could possibly be modulated by T cell and B cell immune responses. These Y-linked genes may be useful prognostic biomarkers and novel treatment targets for HNSCC.

## Supplementary Material

Supplementary Table

Additional supporting information is available in the online version of the article.

## Figures and Tables

**Table 1.** The Correlation of Overall Survival With the Immune Cells Are Different in the HNSCC Patients With High and Low Y-Linked Genes Expression

Molecular analyte metadata	Y-linked genes level					
	High			Low		
	HR	*p* Value	Samples (n)	HR	*p* Value	Samples (n)
Mast.Cells.Resting_low_vs_high	0.51	3.99e–04**	246	1.10	0.48	247
Mast.Cells.Activated_low_vs_high	1.80	1.53e–03**	246	1.00	0.85	247
Lymph.Infiltration.Signature.Score_low_vs_high	0.54	1.12e–03**	246	0.82	0.25	247
B.Cells.Naive_low_vs_high	0.89	0.53	246	0.63	7.84e–03**	247
T.Cells.CD4.Naive low_vs_high	1.80	0.03*	246	1.50	0.08	247
T.Cells.Follicular.Helper_low_vs_high	0.60	7.56e–03**	246	0.88	0.43	247
T.Cells.Regulatory.Tregs_low_vs_high	0.62	0.01*	246	0.83	0.27	247
TCR. Richness_low_vs_high	0.66	0.03*	228	0.89	0.50	217

Abbreviation: HR, hazard ratio.

* *p* < 0.05, significant difference.

** *p* < 0.01, significant difference.
