# Supplementary material for: Y-Chromosome-Linked Genes Are Associated With Sex-Related Head-Neck Squamous Cell Carcinoma Survival
Source: Otolaryngol Head Neck Surg. Author manuscript; Available in PMC 2026 Jul 6. (PMC13334491; doi:10.1002/ohn.421)
Supplement: Supplementary Table [file NIHMS2186401-supplement-Supplementary_Table.docx]

**Supplementary Table 1.** Correlation between Y-linked 9 genes mean expression and male patients survival among different cancers.

|  | Male patients | |
| --- | --- | --- |
| Cancer types | HR | P value |
| Bladder Carcinoma | 0.68 | 0.032 |
| Head-neck squamous cell carcinoma | 0.64 | 0.007 |
| Pancreatic ductal adenocarcinoma | 0.52 | 0.025 |
| Rectum adenocarcinoma | 0.26 | 0.010 |
| Thymoma | 0.00 | 0.021 |
| Stomach adenocarcinoma | 1.68 | 0.010 |
| Kidney renal papillary cell carcinoma | 2.16 | 0.030 |

HR, hazard ratio
